# Early Management, With a Minimal Initial Hospitalization Length, of Major Self-inflicted Rifle Wounds to the Face by a Single Latissimus Dorsi Free Musculocutaneous Flap: A 10-Year Experience

**Published:** 2009-06-09

**Authors:** A. M. Danino, P. G. Hariss, J. M. Servant

**Affiliations:** ^a^ Service de chirurgie plastique, CHU de Dijon Université de Bourgogne, 3 rue faubourg Raines 21000 Dijon, France; ^b^ Service de chirurgie plastique Centre Hopspitalier de l'Universite de Montréal, 1560 rue Sherbrooke-Est, Montréal, Quebec, Canada; ^c^ Service de chirurgie plastique Hopital Saint Louis Université Paris vii 1 avenue claude vellefaud 75010 Paris, France

## Abstract

**Objective:** Severe ballistic injuries to the face create complex, composite defects of 2 facial subunits. These injuries have an extremely high economic impact for the Medicare system. The surgical goal with these patients is to restore basic functions of the face with a rapid morphological improvement. Our hypothesis is as follows: Early restoration of facial segments with a single free multiple island latissimus dorsi flap without primary bone reconstruction can significantly reduce hospitalization time and allow earlier psychiatric therapy with good morphological results. **Surgical method:** (1) Large debridement, bony stabilization with external fixation, and tracheotomy. (2) Definitive early reconstruction of soft tissue with composite free latissimus dorsi-scapular musculocutaneous flap. (3) Several refinements will optimize the results. **Study design:** Retrospective case series of lower- and middle-face composite facial close-range high-energy gunshot wound patients were evaluated. Age, gender, mechanism of injury, anatomic subsites involved, surgical procedures, flaps utilized, complications, functional outcomes, time of tracheotomy closure, hospitalization duration, and beginning of psychiatric treatment were analyzed. **Results:** Twelve defects were gunshot wounds, 12 free latissimus dorsi flaps, and no flap losses. Patients received psychiatric treatment after 22 days (7–29); the tracheotomy was removed in 10 patients with normal alimentation in all cases. Mean hospitalization duration was 21 days. **Conclusions:** Free tissue transfer techniques allow early reconstruction of the soft tissue framework of the face with a single multiple-island flap. Rapid restitution of facial compartments at a soft tissue level can dramatically reduce duration of hospitalization.

According to the energy involved, gunshot wounds are divided in 2 categories: low-velocity injuries (less than 1200 ft/s) and high-velocity injuries (more than 1200 ft/s). The degree of injury and energy involved are proportional to the mass and square of the velocity of the projectile. Self-inflicted rifle wounds to the face are extensive as well as destructive and should be considered as high-energy blast injuries.^1^–^3^ For the purpose of this study, we have defined that a major facial gunshot wound must involve at least 2 anatomic subunits (upper face, middle face, and lower face) with a complete loss of the 3 underlying components (bone, oral lining, and skin).

The classical approach to maxillofacial self-inflicted gunshot injuries includes exposure of all fracture fragments, precise anatomic rigid fixation with immediate bone grafting, and definitive soft-tissue coverage. Early rigid internal fixation remains a gold standard for most of surgeons in high-velocity facial injuries.^4^–^6^ Our hypothesis is that the destruction of facial compartments can be treated with early restoration of facial segments without primary bony reconstruction, thus offering a significant reduction of the hospitalization time, early restoration of speech, and an opportunity to begin early psychiatric treatment. In this study, we intend to present a 10-year experience of major rifle gunshot wounds to the face with early neo segmentation of the facial soft tissues using only a single free flap (multiple skin island musculocutaneous latissimus dorsi free flap) without primary bony reconstruction.

## SURGICAL METHOD

**Figure 1:**
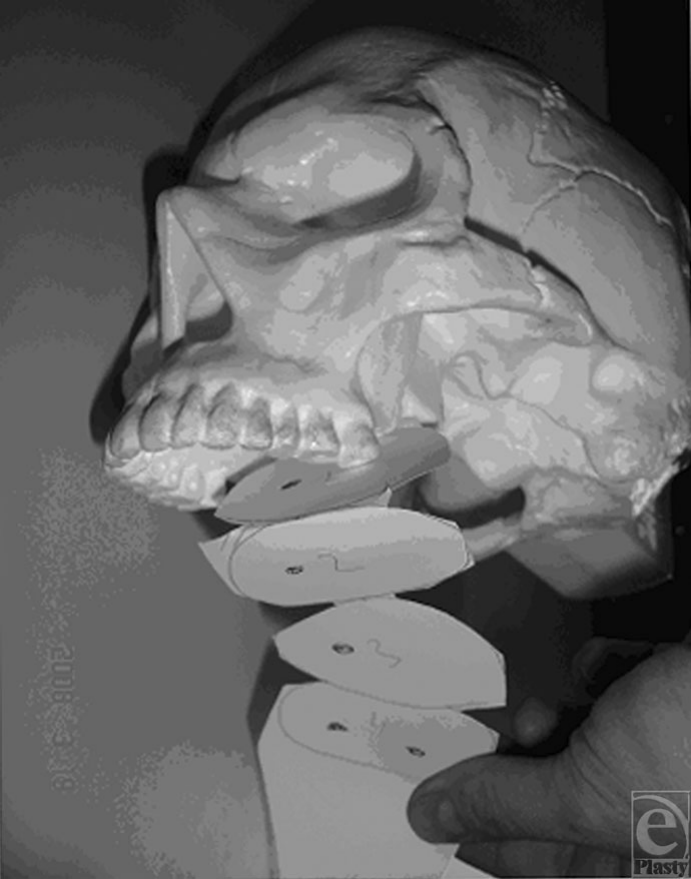
Concept of neo segmentation.

The reparation method has been standardized in 3 phases:

1. Urgently right after hemodynamic stabilization, the patient was prepared and brought to the operating room for debridement of soft tissue and bony stabilization.
  a. First, a tracheostomy secures the airway.
  b. A primary debridement until bleeding tissue is performed allowing for repositioning of the viable structures. The equivocal tissues are left in place until a secondary look is performed. The primary goal of bony stabilization is to preserve facial volume and is performed with an external fixation.
  c. Bilateral tarsorrhaphies are performed to protect the eyes.
  d. A nasogastric tube is inserted allowing enteric feeding.
  e. A dressing is applied with the first layer being nonsticky, followed by acetic acid compresses covering the entire face.
2. The patient is brought to the surgical intensive care unit.
  a. Daily dressing changes are performed and, if required, subsequent surgical debridements as well.
  b. Imaging is performed including 3-dimensional computed tomography reconstruction.
  c. A gastrostomy is performed.
3. The second surgical stage is planned within the first 10 days of the initial trauma.
  a. A large composite latissimus dorsi musculocutaneous flap with multiple skin islands is performed. This flap allows the reconstruction of the neo cavities of the face, especially the oral and nasal ones. The diameters of these cavities are fixed in the vertical dimension during this first stage. The cutaneous island is harvested widely with muscle perforators present only in the first third of the flap. The vascular anastomosis is performed with an operative microscope magnification using 8/0 nylon interrupted sutures. The artery is anastomosed in an end to side fashion on the external carotid, whereas the internal jugular vein is the recipient for the vein.
  b. Sculpturing of the flap: The proximal islands of the flap are used to reconstitute the floor of the nasal cavities. Complete dissection of the different skin islands with a 180° turn in the vertical direction allows reconstruction of the palate. A second separation of a third island allows for a lateral 90° rotation to reconstruct the inside of the cheek and a final twist of 180° allows reconstruction of the external portion of the cheek (Fig 1).
4. Multiple shaping and esthetic refinements are performed in succession without having to transfer new tissues. At least 2 fatty debulkings and shaping procedures are required under general anesthesia to allow the widening of the compartments in the horizontal plane. Alter the initial remodeling, the remaining procedures can be performed under general or local anesthesia.

## STUDY DESIGN

We present a retrospective case series from 1996 to 2006 in 2 university hospitals in which all patients with combined mandible/maxilla defects after high-velocity ballistic injuries were included. Following human subjects institutional review board approval, the charts, radiographs, and patient outcomes were cataloged.

Age, gender, mechanism of trauma, anatomic subsites involved, reconstructive procedures performed, delay to the reconstructive procedure, flaps utilized, complications, hospital stay duration, and the number of surgical procedures were reviewed.

Functional outcomes (swallowing, speech, airway freedom, aesthetics) were also evaluated as follows:

• Speech was determined by the ability of the patient to be intelligible during a telephone conversation.

• Swallowing function was evaluated by the patient diet: regular soft or gastrostomy tube percutaneous enterogastrostomy (PEG)

• Cosmesis was evaluated by the patient and the physician on a visual analog scale of 1 (*worst*) to 10 (*excellent*); the individual scores were averaged to determine the final score.

• Airway function was evaluated by the ability to breathe without a tracheotomy.

## RESULTS

**Figure 2:**
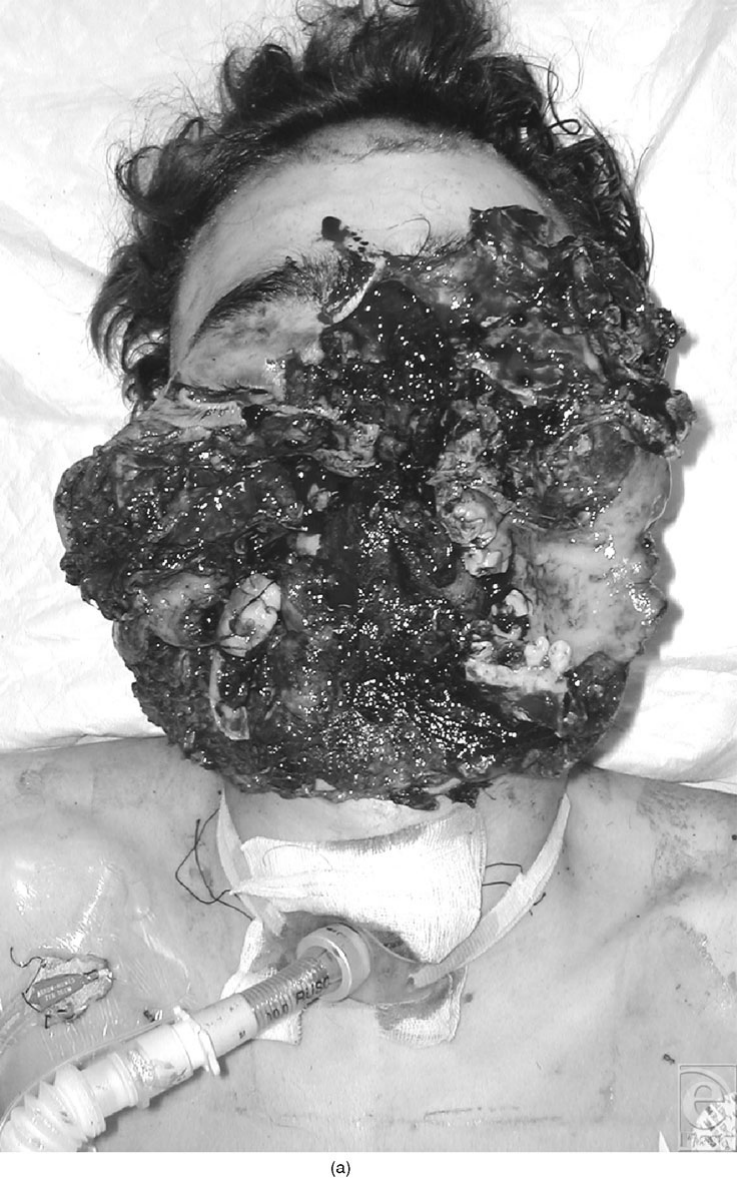

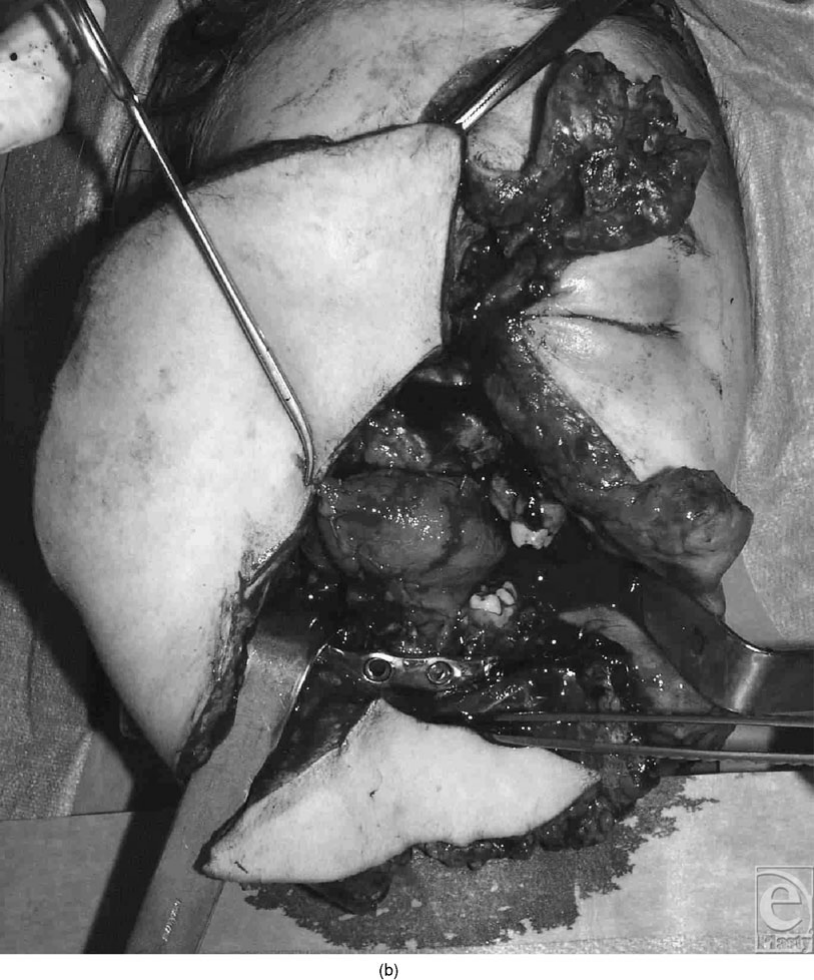

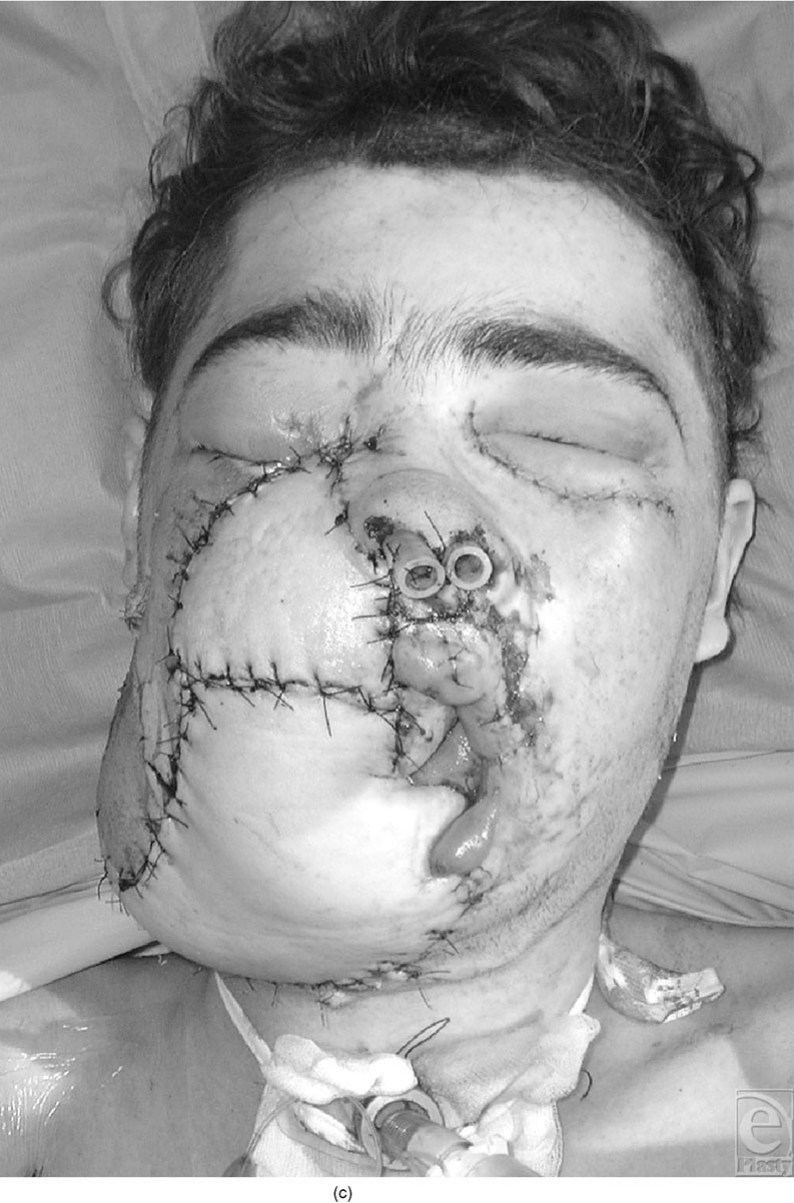

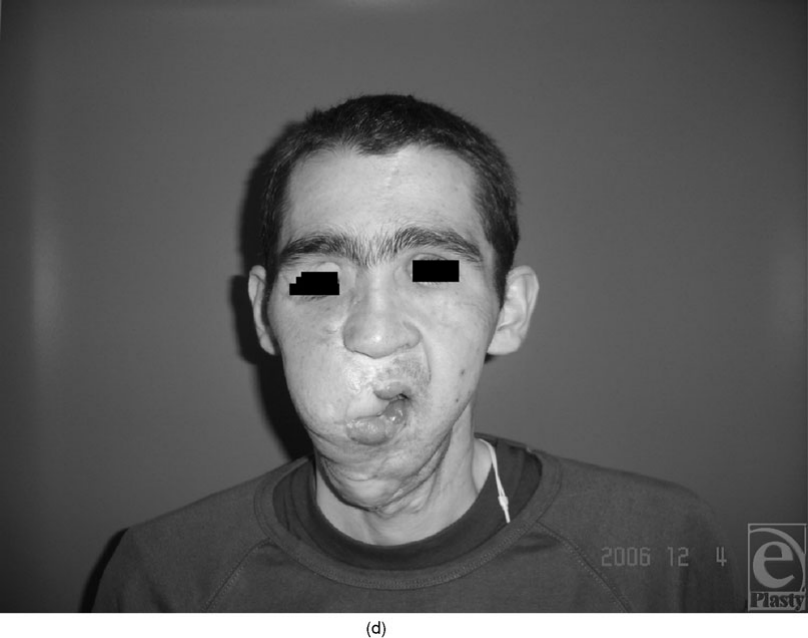
Cases number 2. (a) Preoperative view; (b) neo segmentation; (c) postoperative after 2 local refinements; and (d) after reconstruction by mandible distraction.

**Figure 3:**
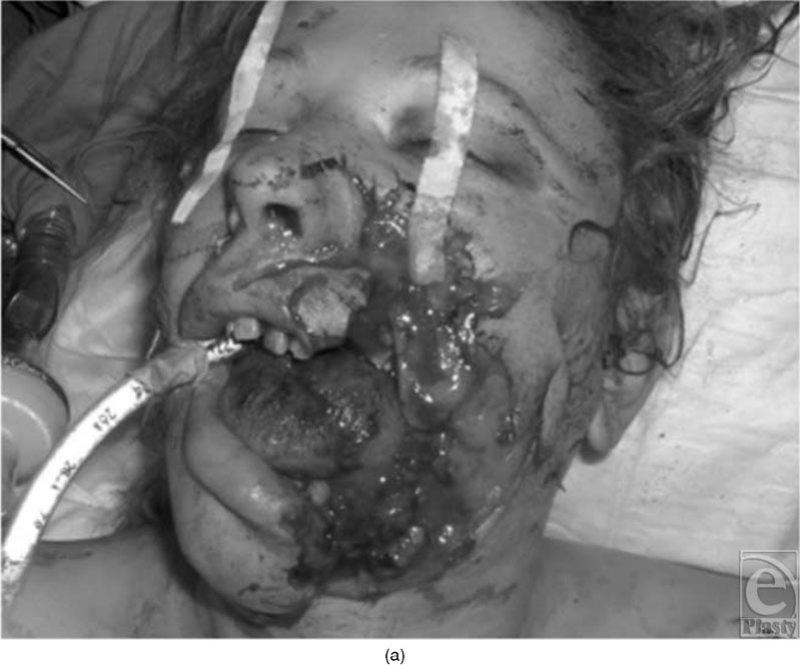

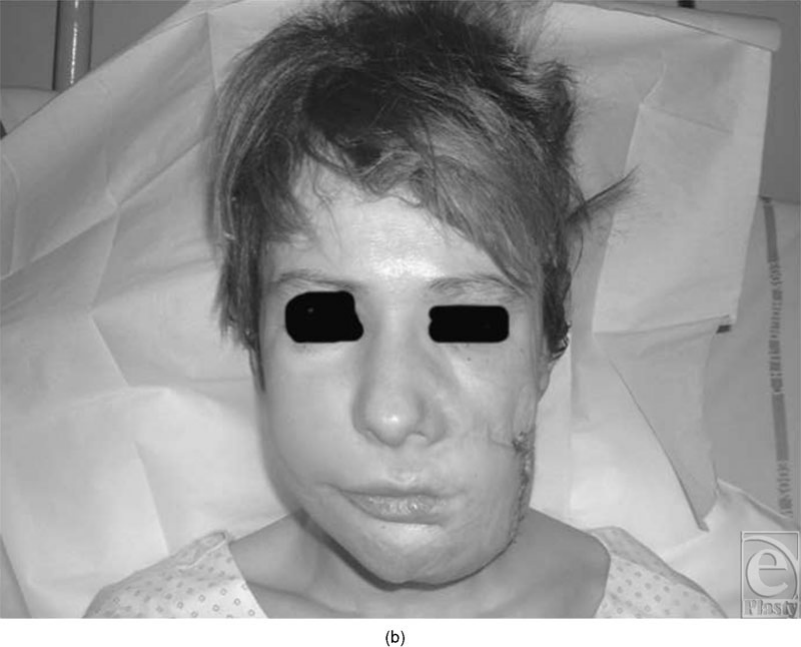
Case number 8. (a) Preoperative view and (b) postoperative view after 1 local refinement.

Demographic information and results of the patient population are listed in Table 1. Two cases are presented as samples of the case series (Figs 2a–2c and 3a and 3b). Twelve patients, including 10 males and 2 females with ages 21 to 56 years, were included in the study. They all presented combined sites and a composite defect at the maxillary/mandible level. None of the patients who attempted suicide had intracerebral lesion. The majority of individuals were in the age group of 20 to 30 years and were predominantly male. All the gunshot wounds were self-inflicted. Follow-up ranged from 1 year to 9 years with a mean of 26 months.

A single, large, musculocutaneous latissimus dorsi flap was performed between 5 and 15 days postinjury. The flaps had anywhere between 2 and 5 skin islands (3 cases with 2 islands, 6 cases with 3 islands, and 3 cases with 5 islands).

One surgical revision was required for the drainage of a hematoma, but there was no necrosis of any part of the flaps. The number of surgical procedures required ranged from 4 to 8 under general anesthesia to shape the different flaps. The most common treatment indicated beyond the initial free tissue transfer was flap debulking, scar revision, bone grafting (secondary), cartilage graft, local flaps, and osteogenic distraction. Approximately twice as many of these phase IV procedures were necessary in patients needing nasal reconstruction to enhance the reconstructive result than any other group.

All patients were able to resume a regular or soft diet at the 32nd day (21–65), and all patients were able to resume intelligible speech over the telephone in an average of 11 days (6–31). The tracheostomy was weaned within 20 days (10–45). Cosmesis evaluated by the patient was on a visual analogical scale on an average of 5.68 (4.5–6.6) and by the physician on an average of 6.3 (5–7.3).

Psychiatric evaluation and treatment were started by the 6th day on average. The hospital stay was on average 21 days (from 17 to 33).

Six patients returned to work, one is in complete social isolation, and four are actively followed in therapy. There was one recurrent suicidal attempt resulting in death.

## DISCUSSION

Facial gunshot wounds are often classified according to the involved area; in 2004, Vayvada et al^7^ described a modified classification including the involved area as well as the tissue composition of the defects. Our case series constitutes the first survey of self-inflicted gunshot wounds to the face with complete destruction of at least 2 subunits with a similar rifle type, missile caliber, and shot distance.

The person who intends to commit suicide typically places the rifle muzzle beneath his chin. Once the trigger is pulled, a jerk may result in reflex extension of the head or turning away from the muzzle. This action alters the intended course of the shot and saves the victim's life, and the charge then explodes into the chin or cheeks, depositing gunpowder, wadding, and shot into the soft tissues while ripping away mucous membrane, bone, cartilage, and skin along its path. Bone and soft tissue damage or loss is often complicated by destruction of the nasal, orbital, oral, and cranial cavities.

We strongly advocate that for this specific subgroup the major problem is a functional deficit caused by the destruction of the facial cavities.

We suggest that facial segmentation can and should be achieved by the use of soft tissue only. This is the only way to reestablish watertight cavities compatible with vital functions of the face. Our strategy is to reconstruct the soft tissue compartments of the face with a single musculocutaneous latissimus dorsi flap, using multiple skin islands to reconstitute the different facial cavities that have been destroyed. Our aim is to restore the soft tissue architecture of the face before the bony one. The bony reconstruction is secondarily required at a later date to support the previously reconstructed compartments.

Suicide attempts with self-inflicted gunshot wounds to the face account for a significant proportion of maxillofacial injuries. Firearms are associated with 66% of homicides and 51% of suicides.^8^ Distressingly, assault-related fatal and nonfatal firearm-related injury rates remain highest among persons 15 to 24 years of age. Traditional methods of treatment self-inflicted gunshot trauma have an extremely high economic impact on the healthcare system: it is the third most costly etiology of injury (first among causes of facial injuries) and the fourth most expensive form of hospitalization.^9^,^10^

Upon literature review, no previous information about initial hospitalization length of facial gunshot injuries was found; therefore, we were forced to compare our series with previous descriptions involving oncological reconstructions composed of 2 or more subunits. When we compared our initial hospitalization length to facial reconstructions of at least 2 subunits, our method gave us a 50% reduction, with a shorter delay to psychiatric evaluation without altering morphological results based on classical visual analog scale measurements (Table 2).

For the past 30 years, the usual treatment was to reconstruct the bony architecture and the soft tissues at the same time. The anatomical subunits were reconstructed using side-by-side restitutions of precisely measured facial units in an illustration of these principles. All poorly vascularized tissues are removed at once, and composite free tissue transfers are immediately used to reconstruct the face unit by unit.^10^–^17^ The major drawback with this technique is the difficulty in adapting all the different subsequent reconstructions. Oftentimes, multiple lengthy procedures are required (up to 14 procedures with 4 free flaps for some authors). This strategy can lead to an extended initial hospitalization time (up to 3 months). The bony distraction theory introduced by McCarthy^18^ and Molina^19^ and more recently by Labbé and colleagues^20^ can provide spectacular results in partial facial destruction but remains hardly applicable to major destructions of 2 facial subunits.

## CONCLUSION

Most of the patients who attempted suicide had psychiatric disorders, which required rapid treatment by a psychiatrist. Our strategy provided a shorter surgical total reconstruction time with a reduction of the initial hospitalization length. This allowed a faster psychiatric evaluation and treatment because function, feeding, and speech were more quickly resumed.

## Figures and Tables

**Table 1:** Demography and results

Patient	M/F	Age, y	days before free Flap	N° of islands in the LD flap	Early complications	Number of surgeries	Removal of tracheotomy	Nutrition	Speech	Cosmetic by the surgeon	Cosmetic by the patient
1	M	55	6	2	0	4	10	40	12	7	6
2	M	21	5	5	1	8	45	45	31	6	6.5
3	M	29	7	3	0	5	19	21	10	6	4.5
4	M	27	10	3	0	5	15	65	11	6	6.6
5	M	33	15	2	0	5	17	33	6	5	4.5
6	M	25	12	5	0	5	20	25	7	7.3	5.5
7	M	45	11	3	0	5	14	16	8	6.5	6.5
8	F	28	14	2	0	4	16	23	10	5.9	5.5
9	M	27	7	3	0	5	18	28	12	6.5	5.5
10	M	40	8	3	0	5	27	22	8	6.6	6
11	M	37	6	3	0	5	9	13	9	7	5.6
12	F	42	8	5	0	8	40	55	30	6	5.5

**Table 2:** Experience with composite defect at the maxillary/mandible level^*^

Source	No. of flaps per patient	No. of cases	Initial hospitalization stay
Vayvada H, Menderes A, Yilmaz M, et al. *J Craniofac Surg*. 2005:16:794–804.	2	5	37
Chen HC, Demirkan F, Wei FC, et al. *Plast Reconstr Surg*. 1999:103:839–45.	2	14	42
Wei FC, Demirkan F, Chen HC, Chen IH. *Plast Reconstr Surg*. 1999:103:39–47.	2	36	45

^*^Comparison to our results: 12 cases/1 free flap/21 days of initial hospital stay.

## References

[1] Barach E, Tomlanovich M, Nowak R (1986). Ballistics: a pathophysiologic examination of the wounding mechanisms of firearms: Part I. J Trauma.

[2] DeMuth WE (1971). The mechanism of shotgun wounds. J Trauma.

[3] Ordog GJ, Wasserberger J, Balasubramaniam S (1988). Shotgun wound ballistics. J Trauma.

[4] Deschler DG, Hayden RE (2000). The optimum method for reconstruction of complex lateral oromandibular-cutaneous defects. Head Neck.

[5] Siberchicot F, Pinsolle J, Majoufre C (1998). [Gunshot injuries of the face. Analysis of 165 cases and reevaluation of the primary treatment]. Annales de chirurgie plastique et esthétique.

[6] Williams CN, Cohen M, Schultz RC (1988). Immediate and long-term management of gunshot wounds to the lower face. Plast Reconstr Surg.

[7] Vayvada H, Menderes A, Yilmaz M (2005). Management of close-range, high-energy shotgun and rifle wounds to the face. J Craniofac Surg.

[8] Morrissey TB, Byrd CR, Deitch EA (1991). The incidence of recurrent penetrating trauma in an urban trauma center. J Trauma.

[9] Brown TD, Michas P, Williams RE (1997). The impact of gunshot wounds on an orthopaedic surgical service in an urban trauma center. J Orthop Trauma.

[10] Wintemute GJ, Wright MA (1992). Initial and subsequent hospital costs of firearm injuries. J Trauma.

[11] Blackwell KE, Buchbinder D, Biller HF, Urken ML (1997). Reconstruction of massive defects in the head and neck: the role of simultaneous distant and regional flaps. Head Neck.

[12] Chen HC, Demirkan F, Wei FC (1999). Free fibula osteoseptocutaneous-pedicled pectoralis major myocutaneous flap combination in reconstruction of extensive composite mandibular defects. Plast Reconstr Surgery.

[13] Inoue T, Harashina T, Asanami S, Fujino T (1988). Reconstruction of the hard palate using free iliac bone covered with a jejunal flap. Br J Plast Surg.

[14] Nakatsuka T, Harii K, Yamada A, Ueda K, Ebihara S (1992). Dual free flap transfer using forearm flap for mandibular reconstruction. Head Neck.

[15] Serletti JM, Coniglio JU, Tavin E, Bakamjian VY (1998). Simultaneous transfer of free fibula and radial forearm flaps for complex oromandibular reconstruction. J Reconstr Microsurg.

[16] Urken ML, Weinberg H, Vickery C (1992). The combined sensate radical forearm and iliac crest free flaps for reconstruction of significant glossectomy-mandibulectomy defects. Laryngoscope.

[17] Wei FC, Demirkan F, Chen HC, Chen IH (1999). Double free flaps in reconstruction of extensive composite mandibular defects in head and neck cancer. Plast Reconstr Surg.

[18] McCarthy JG, Schreiber J, Karp N, Thorne CH, Grayson BH (1992). Lengthening the human mandible by gradual distraction. Plast Reconstr Surg.

[19] Molina F (2004). Mandibular distraction: surgical refinements and long-term results. Clin Plast Surg..

[20] Labbé D, Nicolas J, Kaluzinski E (2005). Gunshot wounds: reconstruction of the lower face by osteogenic distraction. Plast Reconstr Surg.

